# Changes in the Prevalence of Metabolic Syndrome, Its Components, and Relevant Preventive Medication between 2011 and 2018 in the Northeast Hungarian Roma Population

**DOI:** 10.3390/jpm11070595

**Published:** 2021-06-24

**Authors:** Peter Piko, Judit Dioszegi, Zsigmond Kosa, Janos Sandor, Mariann Moizs, Roza Adany

**Affiliations:** 1 MTA-DE Public Health Research Group, University of Debrecen, 4032 Debrecen, Hungary; piko.peter@med.unideb.hu (P.P.); dioszegi.judit@med.unideb.hu (J.D.); 2 Department of Health Methodology and Public Health, Faculty of Health, University of Debrecen, 4400 Nyíregyháza, Hungary; kosa.zsigmond@foh.unideb.hu; 3 Department of Public Health and Epidemiology, Faculty of Medicine, University of Debrecen, 4032 Debrecen, Hungary; sandor.janos@med.unideb.hu; 4 Kaposi Mór Teaching Hospital, 7400 Kaposvár, Hungary; moizs.mariann@kmmk.hu

**Keywords:** metabolic syndrome, prevalence, Roma, preventive medication, targeted public health strategy

## Abstract

Metabolic syndrome (MetS) is a cluster of clinical conditions that poses a major health burden worldwide. In the present study, we investigate the changes in the prevalence of MetS and its components among the Roma in two disadvantaged counties in Northeastern Hungary focusing on a seven-year-long period. The database of the present study is based on cross-sectional surveys of the Hungarian Roma population (aged 20–64 years) conducted in 2011 (*n* = 458) and 2018 (*n* = 374). The increase in the prevalence of MetS itself in the whole Roma population was not found to be significant in the period examined (although it increased from 40.0% up to 46.0%, *p* = 0.080); however, regarding its components, there was a significant increase in the prevalence of central obesity (from 62.7% to 73.3%, *p* = 0.001) and raised blood pressure (BP) or treated hypertension (from 45.2% to 54.5%, *p* = 0.007). These changes were mainly observed in the younger age groups, so the risk for MetS increased significantly in the 20–34 (OR = 1.10, *p* = 0.038) and 35–49 (OR = 1.07, *p* = 0.048) year age groups in the 2018 study population compared the 2011 one. The increasing prevalence of hidden hypertension and, consequently, untreated individuals with raised BP (from 29.6% to 43.5%, *p* = 0.014) among females is quite alarming; therefore, a targeted public health strategy and targeted interventions are desperately needed to prevent further worsening of the current situation.

## 1. Introduction

The Roma population is the largest ethnic minority in Europe (about 10–12 million) and one of the most vulnerable ones; this is why they have been a target of ethnicity-based studies over the past decades. The Roma reside in all countries of the European continent, especially in the Central and Eastern European countries (Bulgaria, Czechia, Slovakia, Romania, and Hungary) [1]. In the 2011 Hungarian census, 316,000 persons identified themselves as Roma [2], but in a research survey, this number was estimated to be approximately 876,000 in 2013 [3].

Generally (regardless of the country where they live), the Roma population can be considered disadvantaged in many aspects of life. The Roma population’s socioeconomic status is generally low [4]; they suffer from and are challenged by unhealthy lifestyles [5,6,7], low education, and high unemployment rates [8]. In addition, their access to the healthcare system is often very limited [9,10].

All these unfavourable factors affect the general health status of the Roma population directly and indirectly [11]. Increased mortality and shorter life expectancy among the Roma are not proven in Hungary (ethnicity is not recorded in the mortality statistics) but can be suspected based on the high prevalence of health risk factors among them. Among the Roma, the frequency of cardiovascular risk factors is higher compared with that among the general population [12,13], and, consequently, their estimated cardiovascular risk is significantly higher, too [14].

In the past decades, many of the European-Union-directed programmes started to accelerate Roma integration and eradicate differences caused by their disadvantaged situation [15,16]. Long-term studies are needed to examine the success of these programmes, but in order to carry out these studies, we need to define the indicators that help us in monitoring. This task is much more difficult to carry out on the Roma population because the data collection for these studies usually faces many challenges [11,17]. The changes in their economic status can be measured by the proportion of active workers, mean household equivalent income, self-assessed financial status, access to a healthcare system, etc., whereas measuring the changes in their health status is much more difficult.

The prevalence of metabolic syndrome (MetS) serves as a valuable indicator to characterise the health status and health risk of a population. MetS is defined by a cluster of interconnected factors that directly and indirectly increase the risk of cardiovascular diseases (CVDs), type 2 diabetes mellitus (T2DM), cancer, non-alcoholic fatty liver disease, dementia, infertility, and other diseases [18]. Metabolic syndrome is defined by the International Diabetes Federation as when a person has abdominal obesity and at least two of the following conditions: raised blood pressure (BP) or treated hypertension, elevated fasting plasma glucose (FPG) concentration or previously diagnosed diabetes mellitus, elevated triglyceride (TG) levels or treated lipid disorder, and reduced high-density lipoprotein cholesterol (HDL-C) levels or treated lipid disorder [19].

Our research group conducted a survey in 2011 in two counties (Hajdú-Bihar (HB) and Szabolcs-Szatmár-Bereg (SSB)) in Northeastern Hungary to define the frequency of MetS and its components in the Roma population and compare it to that in the Hungarian general population. Based on the results of this study, raised fasting plasma glucose (FPG) concentration or formerly diagnosed diabetes mellitus and reduced HDL cholesterol (HDL-C) levels or treated lipid disorder were significantly more frequent in the Roma population, while central obesity and raised blood pressure or treated hypertension were more common in the Hungarian general population. The prevalence of raised triglyceride (TG) levels or treated lipid disorder and MetS did not differ significantly between the study populations [20].

In 2018, our research team conducted a health survey in the same two counties in Northeastern Hungary (HB and SSB) and set a complex database of more than half a million records. Using the databases of the 2011 and 2018 surveys, it was possible to carry out a comparative analysis to examine changes in MetS frequency and its components in the Northeast Hungarian Roma population.

The aims of our present study are: (1) to examine how the prevalence of metabolic syndrome and its components changed among the Northeast Hungarian Roma population between 2011 and 2018; (2) to identify the MetS components and the sex and age groups most affected by the changes; (3) to examine how the proportion of medication related to MetS has changed; and (4) to suggest targeted preventive interventions based on our results.

## 2. Materials and Methods

### 2.1. Study Populations of Roma for Comparison from 2011 and 2018

In our methodology, we used stratified multistage sampling and enrolled Roma participants from Northeast Hungarian counties (Hajdú-Bihar and Szabolcs-Szatmár-Bereg) where most of Roma segregated colonies/settlements are found. These colonies were identified by field workers with Roma origins within the framework of a project by the Hungarian Ministry of Environmental Protection and the University of Debrecen [21]. The ethnicity of individuals was assessed by self-declaration. Only those segregated colonies that consisted of more than 100 individuals were included in the studies.

A questionnaire and a physical-examination-based survey were carried out previously, which provided the 2011 reference population for our present study. In total, 64 Roma colonies were identified, and 40 of them were selected randomly for the study (25 from Hajdú-Bihar and 15 from Szabolcs-Szatmár-Bereg county). For each colony, 25 households were randomly selected, and for each household, all individuals aged 20 years or above were identified, and one of them was randomly selected. After 3 GPs refused to participate, a total of 925 people from the practices of 37 GPs formed the final sample population (22 GPs in Hajdú-Bihar County (22 × 25 individuals) and 15 GPs in Szabolcs-Szatmár-Bereg County (15 × 25 individuals)). A more detailed explanation of the sampling applied and the survey data collected are described in our previous article [20].

In our survey carried out in 2018, 25 colonies were randomly selected based on general practitioners’ (GPs) household lists. In each colony, 20 households were randomly chosen, and then one individual (aged 20–64 years) from each household was enrolled. Face-to-face interviews were conducted at the respondent’s household by university students (with Roma origin) under the supervision of a public health specialist. Each participant was invited to visit a GP for a blood collection and physical examination. The planned sample size was 500 people, of whom 415 signed a consent form to participate in the study. For more details on the study population, see our previous paper [22].

Both surveys included a physical examination and collected information on the medical history and socio-demographic characteristics of each participant. Blood samples were taken and laboratory tests relevant to MetS (fasting glucose levels (FG), triglyceride levels (TG), high-density lipoprotein cholesterol (HDL-C) levels) were performed.

For the study populations, only those subjects were selected who had a complete record of MetS-related phenotype data. These data include age, sex, waist circumference, FPG levels, TG levels, HDL-C levels, and information about antihypertensive, antidiabetic, and lipid-lowering treatments. To avoid the biasing effect of biological differences due to age and sex, the sample populations were matched according to age and sex distribution.

### 2.2. Determination of the Prevalence of Metabolic Syndrome and Its Components

We defined the prevalence of MetS and its components by adopting the International Diabetes Federation definition [23], i.e., a person was considered to have MetS if he or she had central obesity (waist circumference: ≥94 cm for males and ≥80 cm for females—for the Europid population) combined with two or more of the following conditions:

1. raised blood pressure (BP; systolic BP of ≥130 mmHg and/or diastolic BP of ≥85 mmHg) or treatment of previously diagnosed hypertension;
2. raised FG levels (≥5.6 mmol/L) or previously diagnosed type 2 diabetes mellitus;
3. raised triglyceride levels (≥1.7 mmol/L) or specific treatment for this lipid abnormality;
4. reduced HDL-C levels (<1.03 mmol/L in males and <1.29 mmol/L in females) or specific treatment for this lipid abnormality.

### 2.3. Statistical Analyses

All statistical tests were performed using IBM SPSS (version 26, IBM Company, Armonk, NY, USA) software. Prevalence data (sex, metabolic syndrome and its components, age groups, MetS-related treatments) from surveys were compared by χ^2^ test. Subjects were grouped by age (as follows: 20–34, 35–49, and 50–64 years). Adjusted (for age and sex) multivariate logistic regression analyses were applied to examine changes in risk of metabolic syndrome and its components between 2011 and 2018. In generally, the conventional *p* value threshold of 0.05 was applied.

### 2.4. Ethical Statement

All subjects gave their consent to be included in the study before taking part. The study was conducted in accordance with the Declaration of Helsinki, and the protocol was approved by the Ethics Committee of the Hungarian Scientific Council on Health (Reference No.: 8907-O/2011-EKU for the study population from 2011 and 61327-2017/EKU for the study population from 2018).

## 3. Results

### 3.1. Anthropometric and Demographic Characteristics of the Study Populations

Following the selection process by age and sex matching, 458 of the 646 samples from the original 2011 population and 374 of the 415 samples from the original 2018 population were included in the current study. For more details on the characteristics of the study populations, see Table S1.

### 3.2. Parameters Used to Estimate the Prevalence of Metabolic Syndrome and Its Components in the Study Populations

A significant decrease was detected in the average FG levels (5.5 mmol/L in 2011 vs. 5.1 mmol/L in 2018, *p* < 0.001) and systolic blood pressure (from 126.4 mmHg to 123.1 mmHg, *p* < 0.036) from 2011 to 2018 in the sample populations, while a significant increase was found for average HDL-C levels (from 1.2 mmol/L to 1.3 mmol/L, *p* < 0.049), waist circumference (from 90.7 cm to 94.8 cm, *p* = 0.001), and diastolic blood pressure (from 78.8 mmHg to 80.0 mmHg, *p* = 0.032). When examined by sex, a significant change in FG levels (from 6.1 mmol/L to 5.3 mmol/L, *p* = 0.002) was detected only in males. There was a favourable significant change in the average FG levels (from 5.3 mmol to 5.0 mmol/L, *p* < 0.001) for females, while unfavourable change was noted for waist circumference (89.1 cm to 93.8 cm, *p* = 0.001) and diastolic blood pressure (from 78 mmHg to 79.6 mmHg, *p* = 0.026).

The prevalence of antidiabetic therapy also differed significantly between the two study groups (6.8% in 2011 vs. 11.2% in 2018, *p* = 0.024), although these favourable significant changes were only observed in females (from 5.1% to 11.6%, *p* = 0.004), not in males (from 11.0% to 10.3%, *p* = 0.864). The detailed characteristics of the biochemical and physical parameters of the two study populations can be seen in Table 1 and in Table S2A,B differentiated by sex.

### 3.3. The Prevalence of MetS and Its Components in the Study Populations

Central obesity became significantly more frequent (from 62.7% to 73.3%, *p* = 0.001) in the period examined, and similarly, there was significant change in the frequency of raised blood pressure or treated hypertension (from 45.2% to 54.5%, *p* = 0.007). In contrast, there was no significant change in MetS-related lipid parameters, including fasting TG and HDL-C levels, and the prevalence of MetS as such did not increase significantly between 2011 and 2018 (from 40.0% to 46.0%, *p* = 0.080).

In males, there was no significant change in any of the MetS components, and although the prevalence of MetS increased between 2011 and 2018 (from 40.2% to 46.4%), this increase was not statistically significant (*p* = 0.451).

In females, the prevalence of central obesity and raised BP or treated hypertension increased significantly between 2011 and 2018 (from 67.7% to 78.7%, *p* = 0.002; from 42.9% to 53.1%, *p* = 0.012, respectively); nevertheless, the prevalence of MetS did not increase significantly during the same period (from 39.9% to 45.8%, *p* = 0.138).

**Figure 1:**
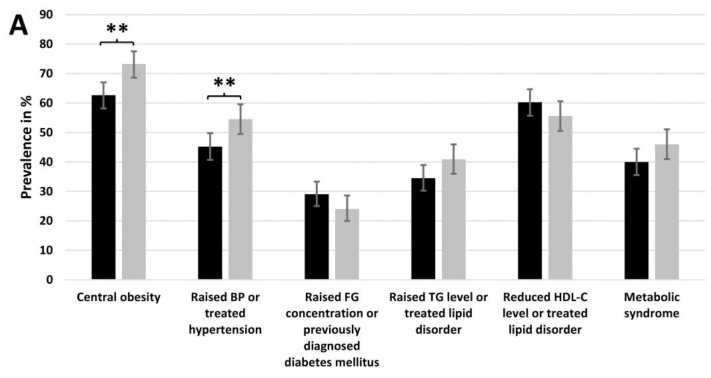

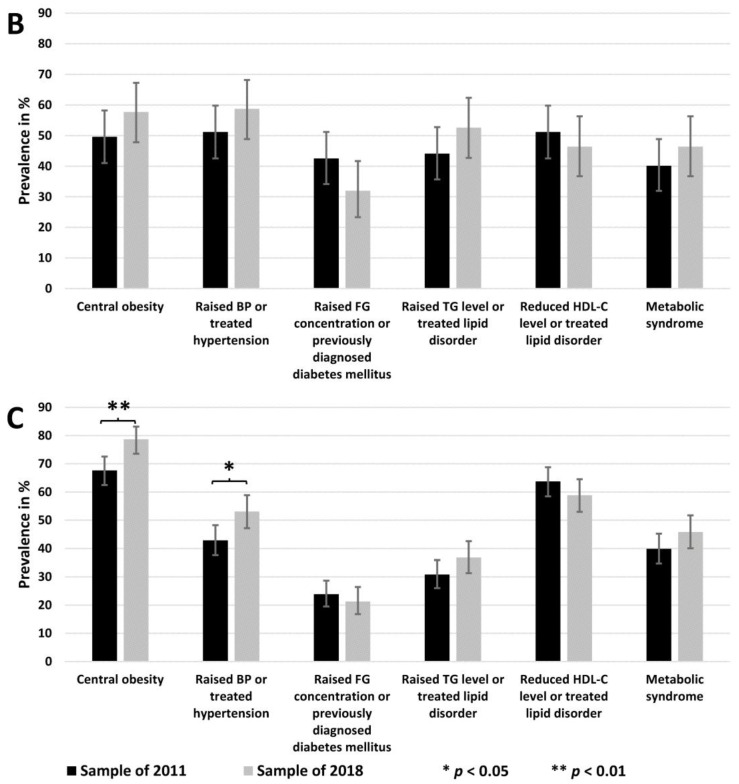
Prevalence of metabolic syndrome and its components among Roma of Northeast Hungary in 2011 and 2018 ((**A**) in the populations studied, (**B**) in males, and (**C**) in females). BP: blood pressure; FG: fasting plasma glucose; TG: triglyceride; HDL-C: high-density lipoprotein cholesterol.

Overall, a favourable change was observed for both sexes for the prevalence of raised FG concentration or previously diagnosed diabetes and the prevalence of reduced HDL-C levels or treated lipid disorder, although the results were not found to be significant, while in the case of central obesity and hypertension, adverse changes were observed (for more details, see Figure 1).

### 3.4. Age-Specific Prevalence of Metabolic Syndrome and Its Components in the Study Populations

An increase in the prevalence of metabolic syndrome was observed in case of the 20–34 and 35–49 age groups between 2011 and 2018, but it was not statistically significant.

In all three age groups examined, there was an unfavourable change in the frequency of central obesity and raised BP or treated hypertension between 2011 and 2018. The prevalence of raised FG concentration or previously diagnosed diabetes mellitus showed a decrease in all three age groups in 2018 compared to the 2011 data. The prevalence of raised TG levels or treated lipid disorder was higher in the 20–34 and 35–49 age groups in 2018 than in 2011, with no change in those over 50 years of age. The prevalence of metabolic syndrome increased in the 20–34 and 35–49 age groups, while a smaller decrease was observed in the 50–64 age group in the 2018 sample population compared to 2011.

**Figure 2:**
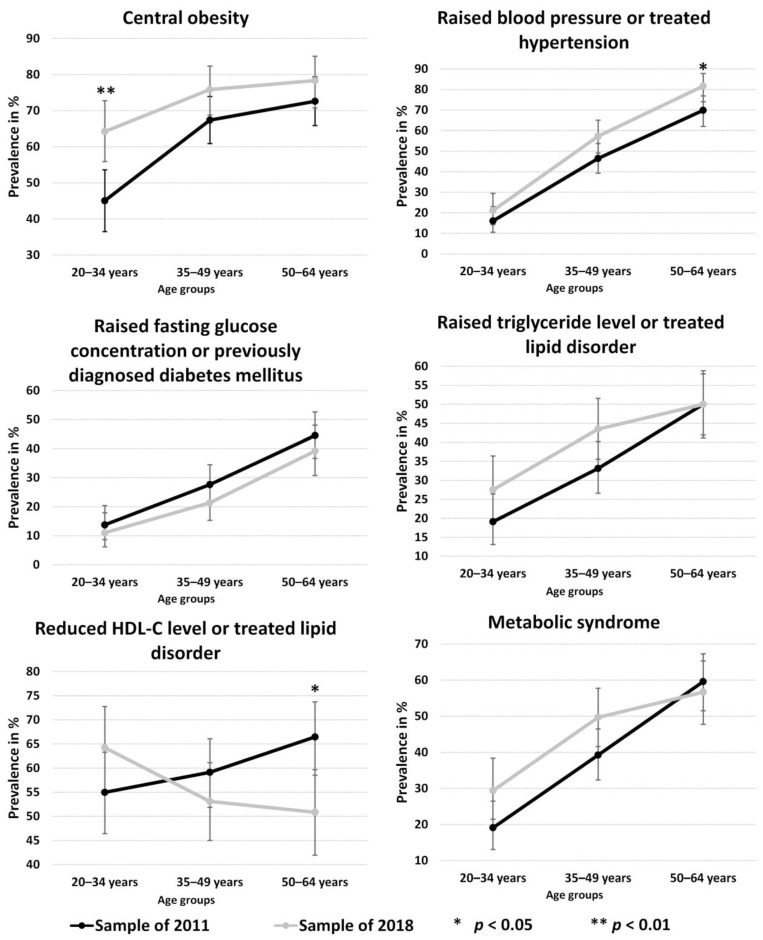
Changes in the prevalence of metabolic syndrome and its components by age group in the Northeast Hungarian Roma population from 2011 to 2018.

Significant differences were observed for the prevalence of central obesity (from 45.5% to 64.2%, *p* = 0.003) in the 20–34 age group, and for raised BP or treated hypertension (from 69.9% to 81.7%, *p* = 0.027) and reduced HDL-C levels or treated lipid disorder (from 66.4% to 50.8%, *p* = 0.010) in the 50–64 age group with opposite directions. The other parameters did not differ significantly between the studied populations. For more details, see Figure 2.

### 3.5. The Change in Risk for the Development of MetS and Its Components between 2011 and 2018

Logistic regression analysis was used to estimate the risk of developing MetS and its components for each age group. The 2011 sample population was used as a reference for the analyses. The risk of central obesity was significantly higher (odds ratio (OR) = 1.13, *p* = 0.002) among the 20–34 year age group in 2018. The risk of raised blood pressure or treated hypertension was significantly higher among the subjects in the 35–49 (OR = 1.07, *p* = 0.037) and 50–64 year age groups (OR = 1.10, *p* = 0.028) in the sample population from 2018. The risk of raised BP or treated hypertension was significantly higher in the 35–49 year age group in 2018 compared to that in 2011.

The risk of metabolic syndrome was significantly higher among the 20–34 (OR = 1.10, *p* = 0.038) and 35–49 year age groups (OR = 1.07, *p* = 0.048) in 2018 compared to 2011. For each age group, the risk of raised FG concentration or previously diagnosed diabetes mellitus decreased between 2011 and 2018, even if not significantly. In the 50–64 year age group, the risk of reduced HDL-C levels or treated lipid disorder (OR = 0.91, *p* = 0.008) decreased among the Roma in the examined period. For more details, see Table 2.

### 3.6. The Change in the Proportion of Those with Untreated Metabolic Syndrome Components in Sample Populations from 2011 and 2018

We examined how the prevalence of individuals not treated for the four treatable components of MetS (listed above) changed in the study populations by sex between 2011 and 2018. The prevalence of untreated individuals with raised FG concentration and/or T2DM significantly decreased (from 76.7% in 2011 to 53.3% in 2018, *p* < 0.001). Contrarily, there was an almost statistically significant increase in the prevalence of untreated individuals with raised blood pressure and/or previously diagnosed hypertension (from 32.9% in 2011 to 42.2% in 2018, *p* = 0.051), and there was no significant change in the prevalence of untreated individuals with raised TG levels between 2011 and 2018 (from 67.7% to 73.9%, *p* = 0.235).

**Figure 3:**
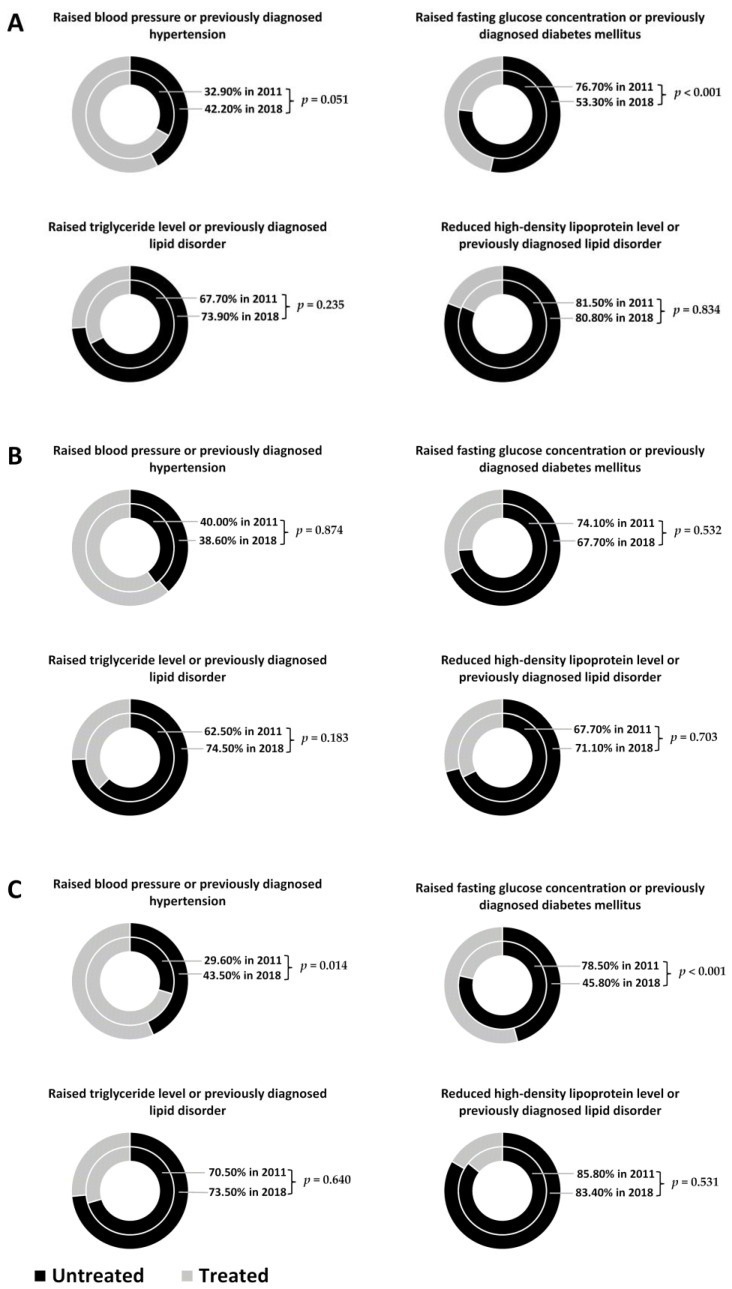
Changes in the prevalence of individuals untreated for the four treatable components of metabolic syndrome (MetS) by study population (**A**) and by sex (male—**B**; female—**C**) from 2011 to 2018.

Data were also examined by sex, and there was no significant change among males in the period tested. Among females, the prevalence of untreated individuals with raised BP was significantly higher in the 2018 sample population than in the 2011 one (29.6% vs. 43.5%, *p* = 0.014), whereas the prevalence of individuals with untreated raised FG concentration and/or diabetes changed favourably from 78.5% to 45.8% (*p* < 0.001) in the period studied. For more details, see Figure 3.

## 4. Discussion

This study reports the changes in the prevalence of MetS and its components between 2011 and 2018 among Roma adults (20–64 years) living in two counties of Northeastern Hungary, where Roma are accumulated.

The increase in the prevalence of MetS between 2011 and 2018 among this population did not reach the level of significance (40.0% vs. 46.0%, *p* = 0.080), although the prevalence of both central obesity and raised BP or treated hypertension showed a significant increase (from 62.7% to 73.3%, *p* = 0.001 and from 45.2% to 54.5%, *p* = 0.007, respectively). The fact that the prevalence of raised TG levels or treated lipid disorder also increased (from 34.5% to 40.9%, *p* = 0.057) cannot be neglected either.

There was no significant increase in the prevalence of MetS and its components in males when examined by sex, while among females, the prevalence of central obesity (from 67.7% to 78.7%, *p* = 0.002) and raised BP or treated hypertension (from 42.9% to 53.1%, *p* = 0.012) increased significantly between 2011 and 2018. It is reasonable to suppose that this phenomenon is a consequence of the fact that diastolic blood pressure changed unfavourably (from 89.1 mmHg to 93.8 mmHg, *p* = 0.001) and the prevalence of antihypertensive treatments barely changed (from 30.2% to 30.0%, *p* = 0.947) among females, while in case of men, neither the systolic nor the diastolic blood pressure changed significantly, and the prevalence of antihypertensive treatments increased by 5.4% (from 30.7% to 36.1%, *p* = 0.397).

An age-specific examination of MetS and its components showed adverse changes in all three studied age groups in terms of central obesity and raised BP or treated hypertension. The risk of metabolic syndrome and raised TG levels or treated lipid disorder was significantly higher in the 20–34 and 35–49 year age groups.

In the 50–64 age group, the risk of reduced HDL-C levels or treated lipid disorder declined between 2011 and 2018. It is a well-known fact that an individual’s HDL-C level is highly defined genetically (about 40–50%) [24], and our previous studies demonstrated that in case of the Roma, the high prevalence of reduced HDL-C levels is clearly due to genetic causes [25,26,27,28]. Thus, the seemingly favourable changes in the 50–64 age group cannot merely be explained by environmental or lifestyle factors, but it is likely that people with low HDL-C passed away due to related diseases at a younger age. Studies have verified that elevated HDL-C levels have a correlation with longevity [29,30,31,32], which might mean that the favourable results in the case of the Roma only show that many of them with reduced HDL-C levels do not even live up to age of 50. The share of individuals with raised BP and raised TG levels also increased in the 20–34 and 35–49 year age groups in 2018 compared to 2011, which may also foreshadow an increased risk of cardiovascular diseases and early death [33].

Even if they were not significant, some favourable changes could also be observed between 2011 and 2018 for the prevalence of raised FG concentration or previously diagnosed diabetes mellitus. The proportion of untreated diabetes declined significantly between 2011 and 2018 (from 76.7% to 53.3%, *p* < 0.001), mainly due to the fact that the number of treated people doubled among Roma females (from 5.1% to 11.6%; *p* = 0.004).

The impact of MetS and its components imposes a significant burden on the healthcare system and the country’s economy. It is further aggravated by the COVID-19 pandemic since it is a well-known fact that MetS and its components are risk factors strongly influencing the progression and prognosis of the disease [34,35,36,37]. Moreover, studies have also shown that ethnic minorities are less likely to follow the Government’s restrictions designed to stop the spread of COVID-19 [38] and tend to have a more critical attitude towards vaccination [39].

In any case, it is necessary to intervene to reduce the frequency of MetS and its components among Roma as in the general population. There are several options available to increase the effectiveness of preventive interventions. Primary health care should be reoriented to preventive services of public health. In Hungary, general medical practice is currently limited to patient care and referral to specialised care. There is a significant lack of screening programs ensuring early detection of special diseases (such as lipid disorders, high blood pressure, and diabetes) and lifestyle counselling to prevent the development of chronic noncommunicable diseases [40]. This is exactly why the increased frequency of MetS and some of its components can be considered even more worrying. In primary care, nutrition counselling is not sufficient, which is supported by the fact that the nutritional habits of the Hungarian general and Roma populations are unhealthy [41]. The lack of preventive services is generally characteristic of Hungarian health care (such as, for example, providing information on cancer screening, measuring anthropometric and laboratory parameters relevant to the development and progression of CVDs and early detection of metabolic syndrome, etc.), and patient willingness to utilise these services is also insufficient [42,43]. Our statement, as we concluded based on observations in model programs we conceptually developed and implemented in cooperation with other members [40,44], that “The future of general practices lays in multidisciplinary teams in which health mediators recruited from the serviced communities can be valuable members, especially in deprived areas” [45], should be emphasised.

An advantage of the study is also a disadvantage, as the comparative analysis of samples from the same region can eliminate the confounding effect of different environmental factors. Another limitation of the study is that the Roma population of the two examined counties cannot be considered representative of the whole Hungarian Roma population; however, since these two counties are also considered disadvantaged from a public health point of view, all preventive interventions should have a much more measurable effect on the health of people living there. Furthermore, in the absence of data, our findings are not adjusted for the effects of changes in lifestyle and dietary habits on the prevalence of MetS and its components in the different sexes and age groups studied.

It is also worth mentioning that the frequency of metabolic syndrome is being studied in many countries around the world, but the changes in its frequency in given intervals are much less studied. Therefore, these types of studies are greatly needed to examine the effectiveness of targeted interventions, the purpose of which is to reduce the frequency of MetS.

## 5. Conclusions

Our results clearly show that the prevalence of metabolic syndrome increased among the Northeastern Hungarian Roma population between 2011 and 2018. This change is mainly seen among females and is most severe in the 20–34 and 35–49 year age groups in both sexes. Stopping this trend would require interventions that focus on the increasing prevalence of central obesity and hypertension in the Roma population.

## Figures and Tables

**Table 1:** Biochemical and physical parameters and the frequency of preventive medication, used to estimate the prevalence of metabolic syndrome in the study populations.

**A**	**Sample from 2011**	**Sample from 2018**	***p*** **-Value**
	**Mean (95% CI)**	**Mean (95% CI)**	
Fasting plasma glucose (mmol/L)	**5.5 (5.3–5.7)**	**5.1 (5.0–5.3)**	**<0.001**
Fasting TG (mmol/L)	1.6 (1.5–1.7)	1.6 (1.5–1.7)	0.274
HDL-C (mmol/L)	**1.2 (1.2–1.2)**	**1.3 (1.2–1.3)**	**0.049**
Waist circumference (cm)	**90.7 (89.1–92.3)**	**94.8 (93.1–96.5)**	**0.001**
Systolic blood pressure (mmHg)	**126.4 (124.6–128.3)**	**123.1 (121.4–124.9)**	**0.036**
Diastolic blood pressure (mmHg)	**78.8 (77.9–79.7)**	**80.0 (78.9–81.0)**	**0.032**
**B**	**Prevalence in %** **(95% CI)**	**Prevalence in %** **(95% CI)**	***p*** **-Value**
Prevalence of antihypertensive treatment	30.3 (26.3–34.7)	31.6 (27.0–36.4)	0.709
Prevalence of antidiabetic treatment	**6.8 (4.7–9.3)**	**11.2 (8.3–14.7)**	**0.024**
Prevalence of lipid-lowering therapy	11.1 (8.5–14.3)	10.7 (7.9–14.1)	0.840

Significant differences in mean or prevalence rates are highlighted in bold; 95% CI: 95% confidence interval. TG: triglyceride; HDL-C: high-density lipoprotein cholesterol.

**Table 2:** The results of multivariate logistic regression models (adjusted by age and sex) estimating the risk for metabolic syndrome and its components in the Roma samples from 2018 compared to those from 2011 by age group.

	20–34 Years		35–49 Years		50–64 Years	
MetS and Its Components	OR (95% CI)	*p*-Value	OR (95% CI)	*p*-Value	OR (95% CI)	*p*-Value
Central obesity	**1.13 (1.04–1.22)**	**0.002**	1.07 (0.99–1.14)	0.085	1.04 (0.96–1.13)	0.373
Raised BP or treated hypertension	1.06 (0.96–1.16)	0.270	**1.07 (1.00–1.14)**	**0.037**	**1.10 (1.01–1.19)**	**0.028**
Raised FPG concentration or previously diagnosed diabetes mellitus	0.97 (0.87–1.08)	0.576	0.96 (0.89–1.03)	0.221	0.97 (0.90–1.04)	0.421
Raised TG levels or treated lipid disorder	1.09 (0.99–1.20)	0.059	**1.07 (1.00–1.14)**	**0.041**	1.00 (0.933–1.07)	0.992
Reduced HDL-C levels or treated lipid disorder	1.06 (0.98–1.14)	0.136	0.97 (0.91–1.03)	0.274	**0.91 (0.84–0.98)**	**0.008**
Metabolic syndrome	**1.10 (1.01–1.20)**	**0.038**	**1.07 (1.00–1.14)**	**0.048**	0.98 (0.91–1.05)	0.523

The sample population from 2011 was used as a reference. Significant differences in odds ratios (OR) are highlighted in bold; 95% CI: 95% confidence interval; BP: blood pressure; FPG: fasting plasma glucose; TG: triglyceride; high-density lipoprotein cholesterol: HDL-C.

## Data Availability

Data available on request due to privacy or ethical concerns.

## Supplementary Materials

The following are available online at https://www.mdpi.com/article/10.3390/jpm11070595/s1, Supplementary Table S1. Anthropometric and demographic characteristics of the study populations, Supplementary Table S2. Biochemical, physical parameters and frequency of preventive medications used to estimate the prevalence of metabolic syndrome in the study populations by sex.
