# Supplementary material for: Changes in the Prevalence of Metabolic Syndrome, Its Components, and Relevant Preventive Medication between 2011 and 2018 in the Northeast Hungarian Roma Population
Source: J Pers Med. 2021 Jun 24;11(7):595. doi: 10.3390/jpm11070595 (PMC8304367; doi:10.3390/jpm11070595)
Supplement: Supplementary file 1 [file jpm-11-00595-s001.zip › jpm-1215600-supplementary/supplementary/Sup_tables/Supplementary Table S1.pdf]

Supplementary Table S1 Main demographic characteristics of the study populations.

		Sample of 2011 Prevalence in % (n)	Sample of 2018 Prevalence in % (n)	*p*-value
Sex	Male	27.7 (127)	25.9 (97)	0.562
	Female	72.3 (331)	74.1 (277)	
Age groups	20-34	28.6 (131)	29.1 (109)	0.974
	35-49	39.5 (181)	38.8 (145)	
	50-64	31.9 (146)	32.1 (120)	
		Mean (95% CI)	Mean (95% CI)	*p*-value
Age (years)		42.6 (41.6-43.7)	42.6 (41.3-43.8)	0.927

95% CI: 95% confidence interval.
