# Supplementary material for: Changes in the Prevalence of Metabolic Syndrome, Its Components, and Relevant Preventive Medication between 2011 and 2018 in the Northeast Hungarian Roma Population
Source: J Pers Med. 2021 Jun 24;11(7):595. doi: 10.3390/jpm11070595 (PMC8304367; doi:10.3390/jpm11070595)
Supplement: Supplementary file 1 [file jpm-11-00595-s001.zip › jpm-1215600-supplementary/supplementary/Sup_tables/Supplementary Table S2.pdf]

Supplementary Table S2. Biochemical, physical parameters and frequency of preventive medication used to estimate the prevalence of metabolic syndrome in the study populations by sex.

	Male			Female		
A	Sample of 2011<br>Mean (95% CI)	Sample of 2018<br>Mean (95% CI)	*p*-Value	Sample of 2011<br>Mean (95% CI)	Sample of 2018<br>Mean (95% CI)	*p*-value
Fasting glucose (mmol/L)	**6.1 (5.6-6.5)**	**5.3 (4.9-5.7)**	**0.002**	**5.3 (5.1-5.5)**	**5.0 (4.9-5.2)**	**<0.001**
Fasting TG (mmol/L)	1.8 (1.6-2.1)	1.9 (1.7-2.1)	0.406	1.5 (1.4-1.6)	1.5 (1.4-1.7)	0.384
HDL-C (mmol/L)	1.2 (1.1-1.2)	1.2 (1.1-1.3)	0.225	1.2 (1.2-1.3)	1.3 (1.2-1.3)	0.146
Waist circumference (cm)	94.7 (91.8-97.6)	97.6 (94.0-101.2)	0.185	**89.1 (87.3-91.0)**	**93.8 (91.9-95.8)**	**0.001**
Systolic blood pressure (mmHg)	131.7 (127.9-135.5)	128.4 (125.0-131.9)	0.328	124.4 (122.4-126.5)	121.3 (119.3-123.3)	0.072
Diastolic blood pressure (mmHg)	80.7 (78.9-82.4)	81.0 (78.9-83.1)	0.600	**78.0 (77.0-79.1)**	**79.6 (78.4-80.8)**	**0.026**
B	Prevalence in % (95% CI)	Prevalence in % (95% CI)	*p*-Value	Prevalence in % (95% CI)	Prevalence in % (95% CI)	*p*-value
Prevalence of antihypertensive treatment (%)	30.7 (23.2-39.1)	36.1 (27.0-45.9)	0.397	30.2 (25.5-35.3)	30.0 (24.8-35.5)	0.947
Prevalence of antidiabetic treatment (%)	11.0 (6.5-17.3)	10.3 (5.4-17.5)	0.864	**5.1 (3.1-7.9)**	**11.6 (8.2-15.7)**	**0.004**
Prevalence of lipid lowering therapy (%)	16.5 (10.9-23.7)	13.4 (7.7-21.2)	0.517	9.1 (6.3-12.5)	9.7 (6.7-13.7)	0.773

Significant differences in mean or prevalence rates are highlighted in **bold**. 95% CI: 95% confidence interval. TG: triglyceride; HDL-C: high-density lipoprotein cholesterol.
